# Reduction of oxidative stress a key for enhanced postoperative recovery with fewer complications in esophageal surgery patients: Randomized control trial to investigate therapeutic impact of anesthesia management and usefulness of simple blood test for prediction of high-risk patients

**DOI:** 10.1097/MD.0000000000012845

**Published:** 2018-11-21

**Authors:** Masahiko Tsuchiya, Kazumasa Shiomoto, Koh Mizutani, Kazuya Fujioka, Koichi Suehiro, Tokuhiro Yamada, Eisuke F. Sato, Kiyonobu Nishikawa

**Affiliations:** a Department of Anesthesiology, Osaka City University Graduate School of Medicine; b Department of Clinical Laboratory, Osaka City University Hospital, Abeno-Ku, Osaka, Japan; c Department of Anesthesia, Osaka Rosai Hospital, Kita-Ku, Sakai, Japan; d Department of Biochemistry, Suzuka University of Medical Science, Suzuka, Mie, Japan.

**Keywords:** d-ROMs, oxidative stress, postoperative complications, propofol, surgical recovery, surgical stress, white blood cell count

## Abstract

**Background:**

Oxidative stress may be an integral determinant of surgical stress severity. We examined whether the preoperative level of derivatives of reactive oxygen metabolites (d-ROMs), an oxidative stress biomarker based on total hydroperoxides in circulating blood, is predictive of increased risk of delayed recovery and complications after surgery, as well as the effects of anesthesia management on postoperative recovery in light of oxidative stress.

**Methods:**

Patients (American Society of Anesthesiologists physical status I-II) scheduled for a radical esophagectomy (n = 186) were randomly selected to receive inhalational sevoflurane (n = 94) or intravenous propofol (n = 92) anesthesia. Preoperative blood d-ROMs level, as well as pre-and postoperative plasma ferric-reducing ability, were analyzed to assess oxidative stress, with white blood cell (WBC) count, C-reactive protein (CRP) level, incidence of severe postoperative complications, and postoperative recovery process within 30 days after surgery also examined in a double-blind fashion.

**Results:**

Postoperative normalization of WBC and CRP was extended in patients with elevated preoperative d-ROMs [WBC versus d-ROMs: correlation coefficient (*r*) = 0.58 *P* < .001; CRP versus d-ROMs: *r* = 0.46 *P* < .001]. Receiver operating characteristics analysis of d-ROMs in relation to incidence of severe postoperative complications revealed an optimum d-ROMs threshold value of 410 UCarr and that patients with ≥410 UCarr had a greater risk of complications as compared to those with lower values (odds ratio = 4.7). Plasma ferric-reducing ability was decreased by 61 ± 185 mmol·l^−1^ (*P* < .001) after surgery, demonstrating development of surgery-related oxidative stress, the magnitude of which was positively correlated with preoperative d-ROMs level (*r* = 0.16, *P* = .043). A comparison of the 2 anesthesia management protocols showed that patients who received propofol, an antioxidant anesthetic, had no postoperative decrease in ferric-reducing ability, lower incidence of severe postoperative complications (7 of 92 versus 18 of 94, *P* = .030, odds ratio = 0.35), and faster uneventful recovery time (WBC normalization days 7.1 ± 5.2 versus 13.6 ± 10.2, *P* < .001) as compared to those who received sevoflurane.

**Conclusions:**

Elevated preoperative blood d-ROMs predicts greater intraoperative oxidative stress and increased postoperative complications with prolonged recovery, thus is useful for identifying high-risk patients for delayed and complicated surgical recovery. Reduction of oxidative stress is vital for enhanced recovery, with control by antioxidants such as propofol a possible solution.

## 1 Introduction

Although safety for patients undergoing a major operation has improved, development of postoperative systemic complications remains a serious concern.^[1–5]^ We and others have demonstrated that excess generation of reactive oxygen species (ROS) leads to an imbalanced oxidative stress status, which has been shown to play an important role in the pathogenic mechanisms of many diseases, including diabetes, ischemic reperfusion injury, and heart disease, as well as smoking-related diseases, cancer, and inflammatory disorders.^[6–12]^ Some of those disorders can be resolved by treatment with an antioxidant agent.^[7–12]^ Furthermore, an investigation of redox status in surgical patients revealed that minimally invasive laparoscopic surgery can reduce ROS generation and surgical stress.^[13]^ Also, use of drugs with antioxidant characteristics, such as propofol, local anesthetics, calcium channel blockers, and steroids, has been reported to have potential to reduce oxidative stress and their administration may improve the postoperative clinical course.^[14,15]^ Thus, a working theory has been developed in which oxidative stress is a key player for determining surgical stress and may influence development of postoperative complications coupled with inflammatory disorders.

Since most ROS effectors, such as hydroxyl radicals and superoxides, are highly unstable and rapidly eliminated in vivo, they are difficult to measure.^[16]^ On the other hand, organic hydroperoxides in circulating blood are relatively stable metabolites of ROS,^[17]^ and can be easily and reliably measured under clinical conditions.^[13,18]^ We have shown that a high preoperative level of blood hydroperoxides in patients undergoing cardiac surgery, analyzed using an assay of blood derivatives of reactive oxygen metabolites (d-ROMs), a new method for measuring total hydroperoxies,^[13]^ is closely related to duration of postoperative mechanical ventilation and length of stay in the intensive care unit (ICU).^[19]^ These findings confirm that d-ROMs function as an oxidative stress biomarker, while they also suggest that an increased d-ROMs level noted at a preoperative examination can reveal patient vulnerability to surgery.

Esophageal cancer surgery is highly invasive and known to be associated with postoperative morbidity and mortality, thus several studies have been conducted to investigate risk factors related to their development.^[20–25]^ Many of those findings indicated that advanced age, severe heart disease, poor performance status, and other similar severe systemic comorbidities were independent risk factors.^[20,23]^ However, few surgical patients are complicated with those factors, as they have generally received careful treatment during the perioperative period, because the significance of those conditions is well recognized and revealed in conventional clinical examinations. Thus, such information regarding risk factors may not have a significant contribution to improvement of patient outcome. Furthermore, other studies have reported scoring systems composed of multiple risk factors,^[21,22,24]^ however, they are overly complicated for clinical use or not applicable in preoperative situations. Since the majority of surgical patients are in relatively good condition,^[26]^ a simple predictor or evaluation method that can be applied to such normal patients is anticipated.

With that background in mind, we speculated that preoperative d-ROMs level could serve as a novel predictor of risk of an eventful postoperative course caused by complications. In this study, we analyzed patients undergoing esophageal surgery who had a good physical condition without serious comorbidities, and determined blood d-ROMs level and plasma ferric-reducing ability as indicators of oxidative stress,^[18,27]^ and also examined white blood cell (WBC) count and C-reactive protein (CRP) level as indicators of postoperative systemic inflammation.^[28,29]^ Postoperative complications and physical recovery were investigated as well to assess the invasiveness of surgery and capacity for recovery from surgery in each patient. Furthermore, as a secondary but comparably important outcome, the effects of anesthesia management (inhalational sevoflurane or intravenous propofol) were examined, especially in regard to the antioxidant activity of propofol.^[15,30]^

## 2 Materials and methods

This study was approved by the Ethics Committee of Osaka City University and registered in a publicly accessible database (UMIN Clinical Trials Registry of the Japan Primary Registries Network: 000007193). After receiving written informed consent, we enrolled normal esophageal cancer patients using the following criteria.

(1) Rated as American Society of Anesthesiologists physical status (ASA-PS) I or II with normal blood biochemical analysis and exercise tolerance ≥4 metabolic equivalents (METS) findings.
(2) No serious systemic comorbidities, such as cardiovascular, respiratory, or metabolic disease.
(3) Scheduled for elective radical surgery for esophageal cancer at Osaka City University Hospital, Japan, a high volume hospital for this type of surgery.
(4) Surgical procedures were performed between 2012 and 2014 under thoracoscopy, including esophageal resection, reconstruction of the gastrointestinal tract with the stomach anastomosed with the esophagus at the neck, and 3-field lymph node dissection.

The patients were randomized by computer to undergo surgery under anesthesia with either inhalational sevoflurane or intravenous propofol. Those with a suspected preoperative infection based on an elevated WBC count on the day of surgery, who underwent an intraoperative change in surgical procedure, or who required a blood transfusion during the operation were excluded. All patients, surgeons, attending ward physicians, and authors who analyzed obtained findings were unaware of the anesthesia group assignment and oxidative stress status of each patient. Anesthesia was administered by anesthesiologists blinded to the study details.

During surgery, anesthesia was maintained with 1% to 3% sevoflurane or 4 to 10 mg·kg^−1^·hr^−1^ propofol with fentanyl and rocuronium, as well as a bispectral index within a range of 40 to 60, and systolic blood pressure and heart rate within 70% to 110% of their pre-induction values.^[31,32]^ Following surgery, the patient was transferred to the surgical ICU under mechanical ventilation, then extubated the next morning and transferred to a general ward.

The level of d-ROMs, a proxy for gross level of organic hydroperoxides level in circulating blood,^[13,19,33]^ was preoperatively analyzed, while plasma ferric-reducing ability was measured both before anesthesia induction and after completion of surgery. These values were determined using an FRAS analyzer (Wismerll Co. Ltd., Tokyo, Japan), as previously reported.^[13,19]^ An advantage of this system is its reliability for analysis of biological clinical samples, even when crude, which is performed with simple and quick procedures.^[34]^ For d-ROMs determination with the system, hydroperoxides are converted to alkoxy and peroxy radicals by a Fenton reaction. Next, the radicals are chemically trapped with chromogen (N, N-diethyl-para-phenylenediamine), leading to formation of corresponding radical cations, which are then identified by absorbance at 505 nm. The d-ROMs level is expressed as Carratelli units (UCarr), in which 1 UCarr corresponds to 0.8 mg·l^−1^ hydrogen peroxide. Plasma ferric-reducing ability, another indicator of oxidative stress, was quantified based on the amount of ferric ion reduced to ferrous ion per unit of plasma by measuring change in absorbance at 505 nm and expressed as units of mmol·l^−1^.^[18,27]^ In addition to those parameters, lactate and plasma thiol (R-SH) levels were analyzed before anesthesia induction and after completion of surgery. Analysis of thiols was performed spectrophotometrically using 5,5’-dithiobis-(2-nitrobenzoic acid) (DTNB) reagent.^[7]^

As parameters of systemic inflammation, the postoperative periods until normalization of WBC count (<9000 mm^−3^) and CRP level (<1.0 mg·dl^−1^) were determined. The first day of normalization was defined as the day when the target value had been normal for 2 consecutive days, or when a normal value was coupled with a near-normal value on the preceding or following day. Our previous clinical observations revealed that period greater than 30 days is sometimes required for CRP levels to completely fall to within the normal limit of ≤0.4 mg·dl^−1^ after esophageal surgery. Therefore, the normal value for CRP normalization was set at ≤1.0 mg·dl^−1^.

The numbers of postoperative days until first ambulation and oral intake, length of hospital stay, and number of patients who developed severe complications were also analyzed. First ambulation was defined as when the patient could continuously walk for at least 10 meters with the help of professional medical staff, and first oral intake was when the patient could first eat gelatin or semi-solid food, as observed by professional medical staff. Severe postoperative complications were defined as respiratory insufficiency requiring mechanical ventilation due to pneumonia or other respiratory disorders, cardiovascular events including lethal cardiac arrhythmias, angina, myocardial infarction and systemic circulatory failure, major esophageal anastomotic leakage, deep wound infection, and systemic inflammation including sepsis, as well as renal failure requiring hemodialysis, need for re-operation, and death within 30 days after surgery. The number of patients who developed at least 1 of those was determined.

In a preliminary study, the correlation between preoperative d-ROMs level and postoperative duration until WBC normalization was shown to have a correlation coefficient value of *r* = 0.31. Power analysis indicated that data from at least 80 patients would be required to detect a correlation with a power of 0.8 and statistical significance level of *P* = .05. Therefore, after taking the 2-group study design and potential dropouts into consideration, we decided that a total of 200 patients, including both the sevoflurane and propofol groups, were required for enrollment in the present study.

Numerical data are expressed as the mean ± standard deviation (SD). For comparisons between groups, continuous data were analyzed using a *t* test or analysis of variance (ANOVA), while categorical data were subjected to analysis by Fisher's exact test and other data were analyzed as appropriate. Receiver operating characteristics (ROC) analysis was performed to determine the optimal cut-off value (with highest sensitivity and specificity) for preoperative d-ROMs level to predict occurrence of severe postoperative complications in our patients. Statistical analysis was performed using JMP 10 (SAS Institute Japan, Tokyo, Japan), Statcel 2 for Excel (OMS Publishing Inc., Tokorozawa, Saitama, Japan), and StatMate 2 and Prism 7 (GraphPad Software, San Diego, CA), as appropriate. The level of statistical significance was set at *P* < .05.

## 3 Results

**Figure 1:**
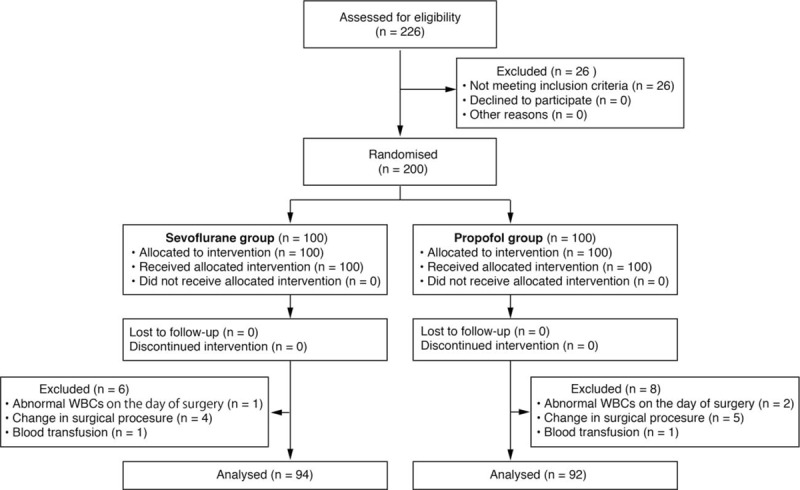
CONSORT diagram of patient recruitment protocol.

Two hundred patients were initially enrolled, of whom 14 were later withdrawn because of a change in surgical procedure, intraoperative blood transfusion, or abnormal WBC count just before surgery. Thus, the remaining 186 patients were analyzed (Fig. 1). Their demographic data are summarized in Table 1.

**Table 1:**
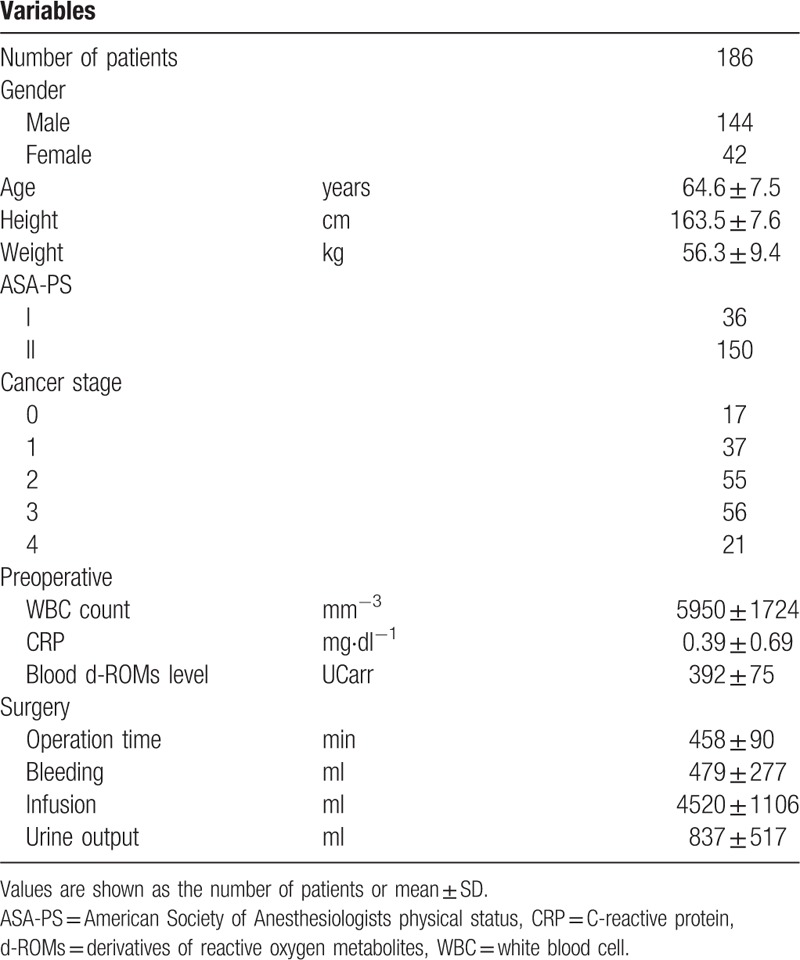
General and surgical characteristics for all esophageal cancer surgery patients.

**Figure 2:**
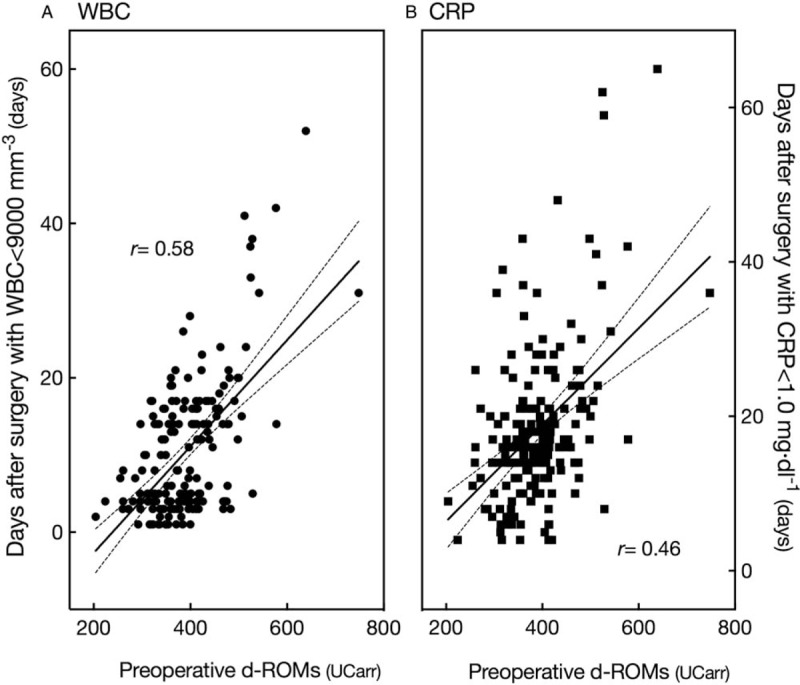
Correlation of preoperative level of d-ROMs, oxidative stress biomarker based on gross level of hydroperoxides in circulation, with (A) postoperative days until normalization of WBC count (<9000 mm^−3^) and (B) CRP level (<1.0 mg·dl^−1^). Solid lines indicate regression lines with a 95% confidence interval (dashed lines) and *r* indicates correlation coefficient. CRP = C-reactive protein, d-ROMs = derivatives of reactive oxygen metabolites, WBC = white blood cell.

**Figure 3:**
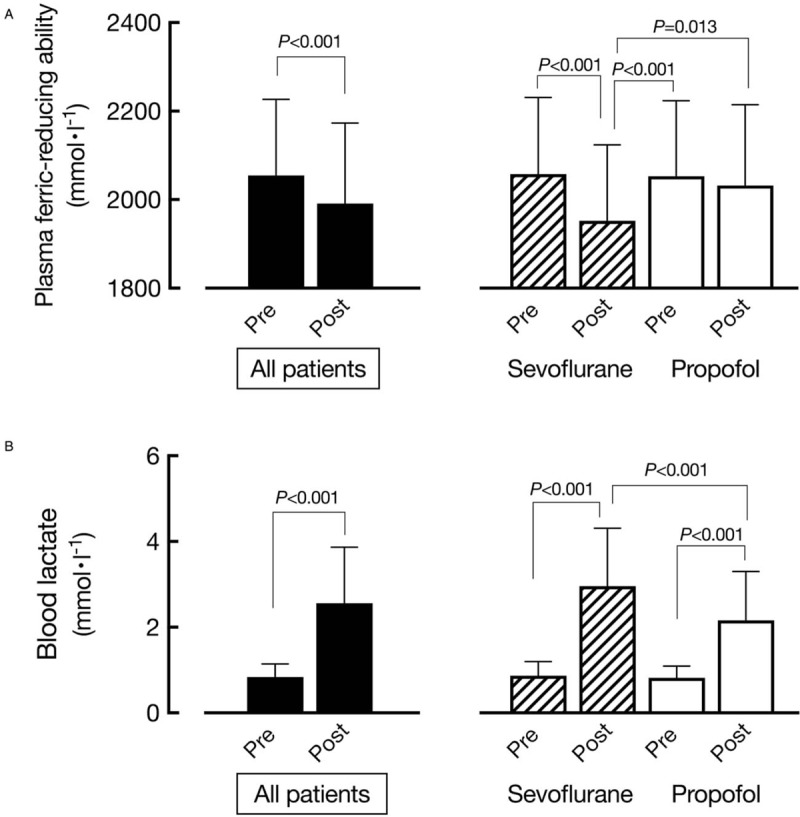
Pre- (pre) and postoperative (post) (A) plasma ferric-reducing ability and (B) lactate levels. Findings shown in left panels were generated using data from all patients (n = 186), while those on the right show comparisons between patients who received sevoflurane (n = 94) and those who received propofol (n = 92) anesthesia (paired t-test or ANOVA, as appropriate). *P* values are shown for significantly different pairs.

There were significant positive correlations of postoperative duration until normalization of WBC count and CRP level with preoperative d-ROMs level (Table 2, Fig. 2) [WBC, correlation coefficient (*r*) = 0.58, *P* < .001; CRP, *r* = 0.46, *P* < .001]. Following surgery, plasma ferric-reducing ability was significantly decreased, as the average postoperative change (postoperative minus preoperative value) was shown to be -61 ± 185 mmol·l^−1^ [*P* < .001, 95% confidence interval (CI): −88 to −34] (Table 2, Fig. 3A left panel). Analysis of the relationship with preoperative d-ROMs level showed that plasma ferric-reducing ability was greatly decreased in patients with high preoperative d-ROMs. Thus, there was a significant negative correlation of postoperative change in plasma ferric-reducing ability with preoperative d-ROMs level (*r* = −0.16, *P* = .043) (Table 2). Since plasma ferric-reducing ability changes in accordance with generation of oxidative stress,^[13,18,27]^ our findings showing that a higher preoperative level of d-ROMs is associated with a greater postoperative decrease in plasma ferric-reducing ability has great importance for understanding the nature of oxidative stress that occurs during surgery. Furthermore, following surgery, lactate level was significantly increased by 1.7 ± 1.2 mmol·l^−1^ (*P* < .001, 95% CI: 1.5 to 1.9) (Table 2, Fig. 3B left panel), while plasma thiols were significantly decreased by 21 ± 67 μmol·l^−1^ (*P* < .001, 95% CI: −31 to −10) (Table 2). In contrast, there was no significant relationship seen between operation time and decreases in plasma ferric-reducing ability or lactate level.

**Table 2:**
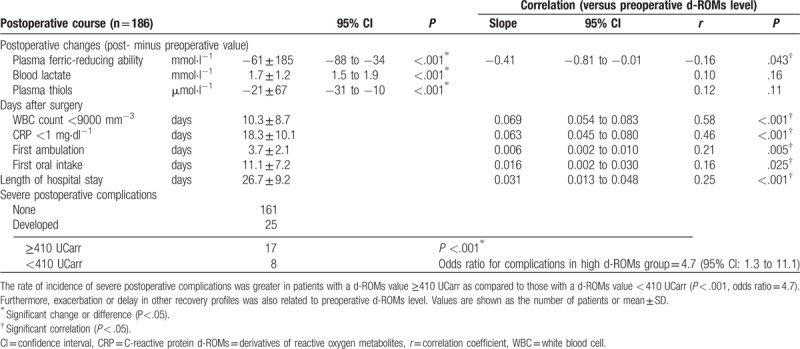
Postoperative recovery findings for all esophageal cancer surgery patients.

**Figure 4:**
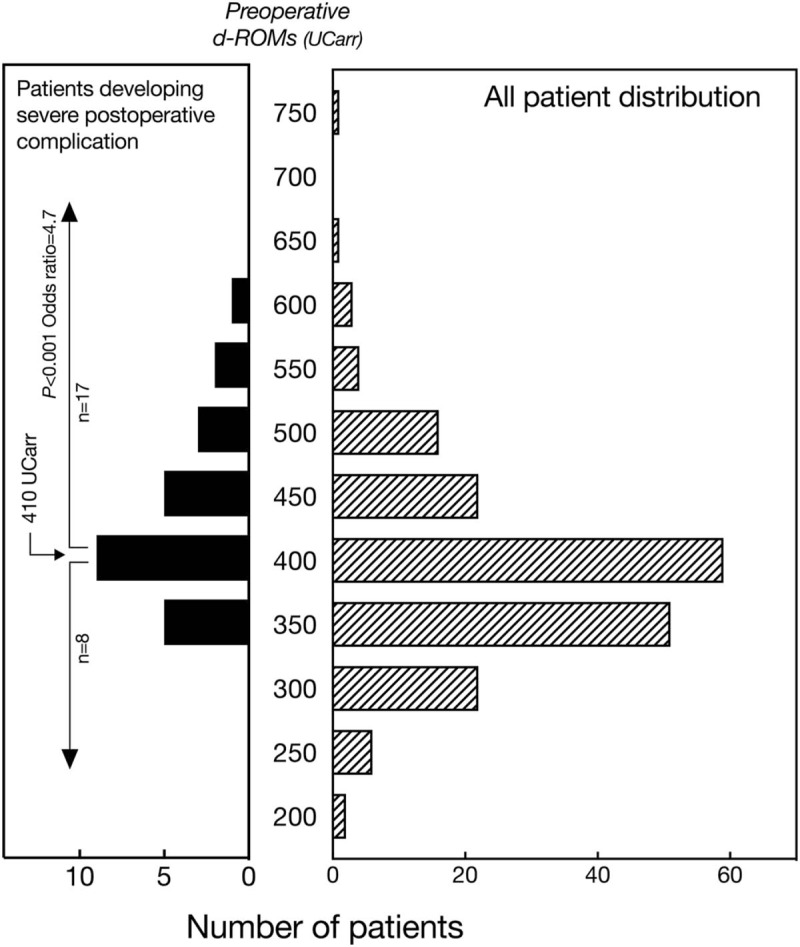
Distribution of preoperative levels of d-ROMs in all patients (n = 186) and those who developed severe postoperative complications (n = 25). The odds ratio for risk of developing a severe complication in patients with ≥410 UCarr was 4.7 (95% CI: 1.3 to 11.1). Severe postoperative complications were defined as respiratory insufficiency requiring mechanical ventilation due to pneumonia or other respiratory disorders, cardiovascular events including lethal cardiac arrhythmias, angina, myocardial infarction and systemic circulatory failure, major esophageal anastomotic leakage, deep wound infection, and systemic inflammation including sepsis, as well as renal failure requiring hemodialysis, need for re-operation, and death within 30 days after surgery. d-ROMs = derivatives of reactive oxygen metabolites.

Figure 4 shows the distribution of all patients as well as those who developed severe postoperative complications according to preoperative d-ROMs level, which indicated that complications developed in association with elevated d-ROMs. The mean d-ROMs level was 392 UCarr, which corresponded well to previously reported findings for the same age group.^[33]^ ROC analysis of d-ROMs level in relation to the incidence of severe postoperative complications revealed that the optimum threshold value for d-ROMs was 410 UCarr (sensitivity, 71.4%; specificity, 71.6%). When the patients were divided into 2 groups based on this cutoff value, the incidence of severe complications in the group with a high level of d-ROMs was significantly greater as compared to the group with a low level (*P* < .001), with an odds ratio of 4.7 (95% CI: 1.3 to 11.1) (Table 2, Fig. 4). Moreover, 3 patients in the high d-ROMs group died, whereas there were no deaths in the low d-ROMs group.

The number of postoperative days until ambulation and oral intake, and length of hospital stay are shown in Table 2. Each of these parameters had a positive though weak correlation with preoperative d-ROMs level [days until ambulation: *r* = 0.21, *P* = .005; days until oral intake: *r* = 0.16, *P* = .025; length of hospital stay: *r* = 0.25, *P* < .001]. These findings indicate that a higher preoperative d-ROMs level is associated with greater delay in postoperative recovery, while a lower level predicts early recovery.

In the second outcome measurement, comparisons between the sevoflurane (n = 94) and propofol (n = 92) anesthesia groups found no differences in regard to either patient or surgical characteristics (Table 3). On the other hand, the numbers of days until WBC and CRP normalization were lower in the propofol group. Furthermore, a postoperative decrease in plasma ferric-reducing ability was observed in the sevoflurane group but not in the propofol group (Table 3, Fig. 3 right panel), while the increase in lactate and decrease in thiol levels were both smaller in the propofol group, indicating that use of propofol anesthesia resulted in reduced surgical oxidative stress. Furthermore, days until ambulation and oral intake, as well as length of hospital stay were all significantly reduced in the propofol group as compared to the sevoflurane group, while the incidence of severe postoperative complications was also significantly lower in patients who received propofol (Table 3) [*P* = .030; odds ratio = 0.35, 95% CI: 0.14 to 0.85].

**Table 3:**
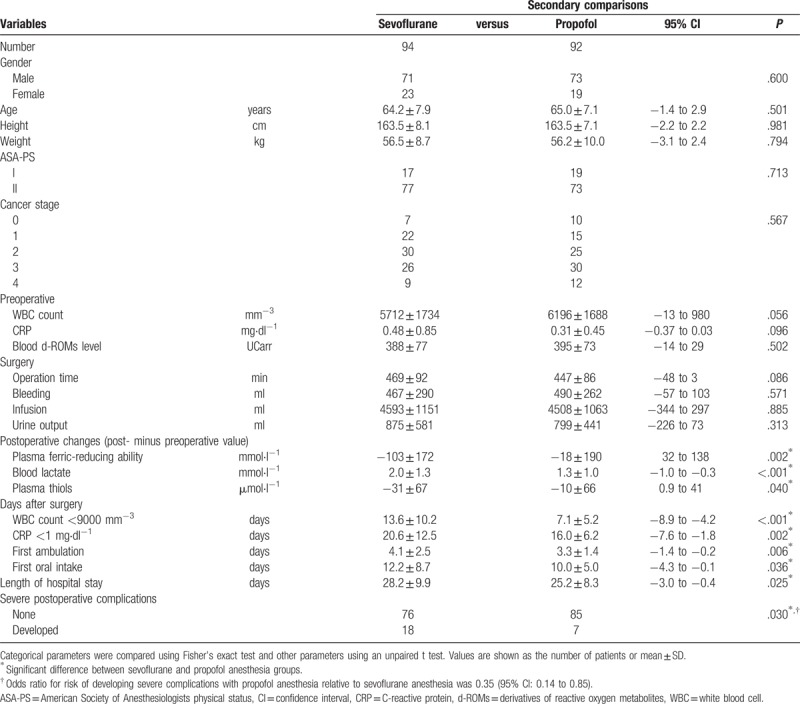
Comparison of patient characteristics and postoperative course between sevoflurane and propofol anesthesia groups.

## 4 Discussion

We analyzed patients who underwent esophageal cancer surgery and found that a higher preoperative level of d-ROMs was closely associated with delay in postoperative normalization of WBC count and CRP level. d-ROMs are an oxidative stress biomarker and used as a proxy for total level of blood hydroperoxides,^[13,19,33]^ with WBC count known to reflect systemic inflammation and widely used clinically as an index of postoperative recovery,^[29]^ and CRP level known to correlate well with the magnitude of surgical stress.^[28]^ Thus, normalization of those factors following esophageal cancer surgery indicates resolution of systemic inflammatory status that appears in response to the surgical procedure, though that response is dependent on oxidative stress status in individual patients. In addition, a higher preoperative d-ROMs level was shown to be associated with longer postoperative periods until ambulation and oral intake, and length of hospital stay. Together, these findings indicate that measurement of preoperative d-ROMs can be useful to predict the recovery profile of individual patients after surgery.

A decrease in plasma ferric-reducing ability indicates a concomitant increase in oxidative stress.^[13,18,27]^ Thus, such a decrease following esophageal cancer surgery shows that the procedure generated oxidative stress, as confirmed by a postoperative decrease in plasma thiols, known to be a major determinant of total antioxidant activity in vivo.^[7,35]^ Moreover, the amount of decrease in plasma ferric-reducing ability was found to be correlated with preoperative d-ROMs level, that is, patients with higher levels of preoperative d-ROMs were subjected to greater surgical oxidative stress. An increase in lactate following surgery may indicate some involvement of ischemia-reperfusion injury in surgical oxidative stress.^[36]^

Our results also showed that patients with a higher preoperative d-ROMs level had a greater incidence of severe postoperative complications including death as well as delay in recovery. Since most of such complications are inextricably related to systemic inflammatory response, the increased incidence noted in the high d-ROMs group of patients, who showed prolonged inflammation, appears to be a reasonable finding. A reliable preoperative method of risk assessment for delayed recovery and complications enables personalized intensive treatment with close monitoring during the perioperative period, or even consideration of non-surgical treatment, which may provide great clinical benefits to patients and medical staff, as well as cost benefits.

Since d-ROMs level provides only a partial view of oxidative stress status, its increase is not directly related to disease onset or specific clinical manifestations. Nevertheless, an increase in that level, which reflects increased oxidative degeneration of lipids and other cellular components, indicates that the balance between oxidative stress and the antioxidant defense system is skewed towards the former.^[17,18]^ Hence, in patients with a higher d-ROMs level, antioxidant reserve is decreased and, when exposed to invasive stress such as from physical, chemical, or biological sources, dysfunction of the elimination system for toxic ROS can occur or worsen, as oxidative degradation products further activate neutrophils to generate excess ROS.^[37]^ Such a vicious cycle may contribute to development of postoperative complications and delayed recovery.

That notion is supported by our secondary findings showing that propofol, which has an antioxidant activity,^[13,15,30]^ enhanced postoperative recovery by decreasing oxidative stress in those patients who received it as an anesthetic agent. The present findings suggest that in patients with a low level of preoperative d-ROMs, both types of agents (i.e., sevoflurane and propofol) can be safely used, though propofol is preferred for those with a high preoperative d-ROMs value. Although the interventional effect is not large, the resulting improvement in surgical recovery may be quite beneficial for both the patient as well as in daily clinical practice. The effects of anesthesia were previously considered to be generally unrelated to those of interventional treatment, though more recent studies of anesthesia techniques and their effects on long-term survival, as well as cancer recurrence in patients following cancer surgery, have revealed that anesthesia itself may be an important component for determination of surgical outcome.^[38–40]^ The present findings support these new perspectives regarding the expanding role of anesthetic treatment.

There are some limitations to our findings and conclusions. It has been reported that myocardial ischemic damage from cardiac surgery is reduced by use of volatile anesthetics via a preconditioning mechanism,^[41]^ indicating that application of sevoflurane might be advantageous for reduction of oxidative stress. On the other hand, the ineffectiveness of volatile anesthetics for cardiac surgery patients has also been reported,^[42,43]^ and the preconditioning effects remain controversial. In addition, maintenance of near normal immune function following use of propofol anesthesia has been shown,^[44–46]^ while an increase in interleukin-6, as well as other immune dysfunctions may occur after use of inhaled general anesthetics.^[46–48]^ Propofol has anti-inflammatory properties^[49]^ and surgical site infections were reported to be suppressed with use of propofol as compared to sevoflurane.^[50]^ Thus, such effects on immune function may have influenced the present results. For greater understanding of oxidative stress status and the effects of antioxidant treatment in patients undergoing surgery, additional investigations are necessary.

In conclusion, patients who undergo esophageal cancer surgery with a higher preoperative level of d-ROMs, an oxidative stress biomarker based on gross level of hydroperoxides in circulation, are subjected to greater surgical oxidative stress, resulting in delayed recovery following the operation with a prolonged inflammatory period as well as severe complications, as compared to those with a lower preoperative level. Preoperative measurement of d-ROMs may be important for predicting the risk of aggravating postoperative events in patients undergoing esophageal cancer surgery. Oxidative stress is an important factor related to surgical stress and postoperative recovery, thus its reduction by anesthesia management with a clinical agent possessing an antioxidant characteristic such as propofol may be effective for improving surgical recovery.

## Author contributions

**Conceptualization:** Masahiko Tsuchiya, Koh Mizutani, Eisuke F. Sato.

**Data curation:** Kazumasa Shiomoto, Masahiko Tsuchiya.

**Formal analysis:** Kazumasa Shiomoto, Kazuya Fujioka, Eisuke F. Sato.

**Funding acquisition:** Masahiko Tsuchiya.

**Investigation:** Kazumasa Shiomoto, Eisuke F. Sato.

**Methodology:** Masahiko Tsuchiya, Koichi Suehiro, Tokuhiro Yamada, Eisuke F. Sato.

**Project administration:** Masahiko Tsuchiya.

**Resources:** Kazumasa Shiomoto, Masahiko Tsuchiya.

**Supervision:** Koh Mizutani, Kazuya Fujioka, Kiyonobu Nishikawa.

**Validation:** Masahiko Tsuchiya, Koh Mizutani.

**Writing – original draft:** Masahiko Tsuchiya.

**Writing – review & editing:** Masahiko Tsuchiya, Koh Mizutani.
